# Impaired Spleen Formation Perturbs Morphogenesis of the Gastric Lobe of the Pancreas

**DOI:** 10.1371/journal.pone.0021753

**Published:** 2011-06-30

**Authors:** Andreas Hörnblad, Anna U. Eriksson, Elisabeth Sock, Robert E. Hill, Ulf Ahlgren

**Affiliations:** 1 Umeå Centre for Molecular Medicine, Umeå University, Umeå, Sweden; 2 Institut für Biochemie, Emil-Fischer-Zentrum, Universität Erlangen-Nürnberg, Erlangen, Germany; 3 Medical Research Council, Human Genetics Unit, Western General Hospital, Edinburgh, United Kingdom; Childrens Hospital Los Angeles, United States of America

## Abstract

Despite the extensive use of the mouse as a model for studies of pancreas development and disease, the development of the gastric pancreatic lobe has been largely overlooked. In this study we use optical projection tomography to provide a detailed three-dimensional and quantitative description of pancreatic growth dynamics in the mouse. Hereby, we describe the epithelial and mesenchymal events leading to the formation of the gastric lobe of the pancreas. We show that this structure forms by perpendicular growth from the dorsal pancreatic epithelium into a distinct lateral domain of the dorsal pancreatic mesenchyme. Our data support a role for spleen organogenesis in the establishment of this mesenchymal domain and in mice displaying perturbed spleen development, including *Dh* +/−, *Bapx1*−/− and *Sox11−/−*, gastric lobe development is disturbed. We further show that the expression profile of markers for multipotent progenitors is delayed in the gastric lobe as compared to the splenic and duodenal pancreatic lobes. Altogether, this study provides new information regarding the developmental dynamics underlying the formation of the gastric lobe of the pancreas and recognizes lobular heterogeneities regarding the time course of pancreatic cellular differentiation. Collectively, these data are likely to constitute important elements in future interpretations of the developing and/or diseased pancreas.

## Introduction

**Figure 1:**
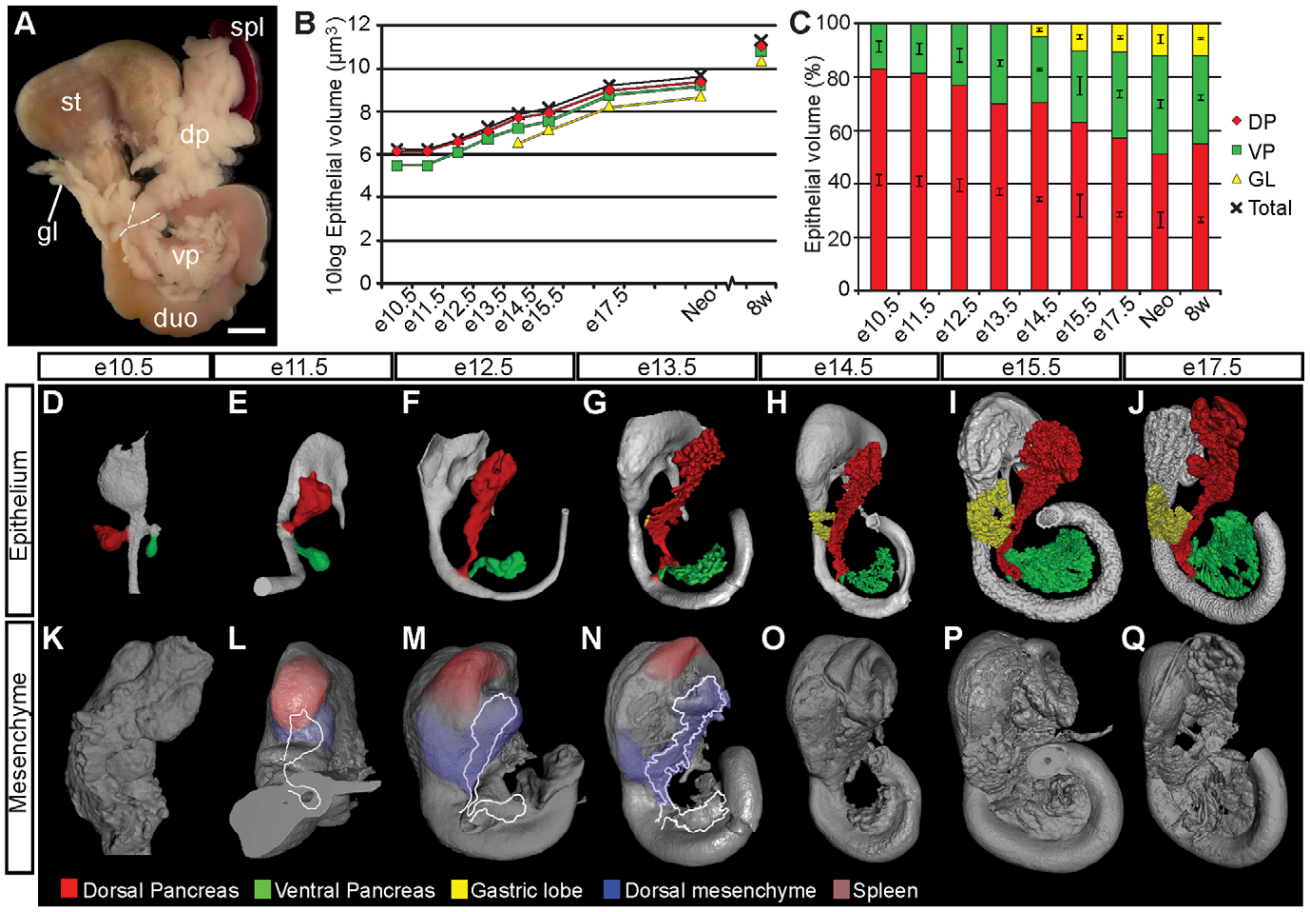
The gastric lobe constitutes a significant portion of the mouse pancreas and is formed by perpendicular growth from the stalk of the dorsal pancreas into a lateral mesenchymal domain positioned in close proximity to the pyloric sphincter and pyloric antrum. (**A**) Photomicrograph of a gut segment including the stomach, duodenum, spleen and pancreas from a C57Bl/6 mouse at 8 weeks. (**B**) Graph depicting the 10log values of the epithelial volume of the DP (yellow diamond), VP (green square), GL (yellow triangle) and the entire pancreas (black cross) between e10.5 and 8 weeks. (**C**) Relative lobular volumes between e10.5 and 8 weeks. From e15.5 onwards, the gastric lobe constitutes more than 10% of the pancreatic epithelium. DP: red; VP: green; GL: yellow. Data in (**B**, **C**) is derived from analyses of C57Bl/6 mice. Values in (**C**) are given ± SEM. (**D** through **Q**) OPT generated iso-surface reconstructions of gut segments including the stomach, duodenum, spleen and pancreas between e10.5 to e17.5 based on the signal from E-cadherin antibodies (epithelium, **D** to **J**) and the signal from tissue autofluorescence (mesenchyme, **K** to **Q**). The condensation and migration of primordial spleen cells in an anterior direction results in the formation of a lateral mesenchymal domain which provides the template for growth from the stalk of the dorsal pancreatic epithelium. (**D** to **J**) The dorsal, ventral and gastric epithelium have been pseudocolored red, green and yellow respectively. (**L** to **N**) The dorsal mesenchyme and the spleen primordium have been pseudocolored blue and pink respectively. The specimens in (**D**–**Q**) are not depicted to scale. Scale bar in **A** is 3 mm.

The mouse pancreas has been extensively studied as a model for embryonic patterning and branching-morphogenesis. With the development of transgene technology the mouse has also become the outstanding system for research on the genetics underlying pancreas related diseases, including diabetic disorders and cancer. An accurate description of normal pancreas development and constitution is hence indispensable to fully appraise potential aberrations relating to these research areas. Traditionally, the pancreas is described to form by a dorsal and ventral evagination of the gut tube epithelium resulting in the formation of a dorsal (DP) and ventral (VP) pancreatic bud. During subsequent development these buds grow, branch, differentiate and eventually fuse to form the adult exocrine and endocrine compound gland (for reviews see [1], [2], [3]). A number of signalling molecules expressed in the pancreas-associated mesenchyme are important for induction of the pancreatic program and subsequent growth and differentiation of the pancreatic epithelium [1], [2]. However, the molecular mechanisms directing later pancreatic morphogenesis are poorly understood. The gastric lobe (GL) of the pancreas ([4] and Figure 1A) is conserved in a number of rodent species including hamster, rat and mouse, and has been suggested to correspond to the auricle or “ear of the pancreas” in humans [5]. Given its distinct spatial localization and significant contribution to the overall pancreatic mass (Figure 1A–C), surprisingly few records of this structure are to be found in the literature and very limited information regarding its embryonic development has been presented. It has been demonstrated that pancreatic mesenchymal morphogenesis is independent on growth of the pancreatic epithelium and the pancreatic mesenchyme has been suggested to provide patterning information for pancreatic branching [6], [7]. A recent study addressing epithelial dynamics during pancreatic branching morphogenesis outlined the morphogenesis of the GL epithelium during development [6]. However, limited information exists regarding the gross mesenchymal morphogenesis during pancreas development. We have previously demonstrated the possibility to perform detailed, high contrast, studies of the developing pancreatic epithelium and its associated mesenchyme by optical projection tomography (OPT) [8], [9]. In this study, we provide a quantitative and three-dimensional (3D) description of pancreas development and investigate the morphological events underlying the formation of the GL [10]. Based on analyses of normal and transgenic animals, we propose a model in which proper initiation of spleen formation is key for the formation of this prominent feature of the rodent pancreas.

## Results

### Spatial and quantitative dynamics of pancreas morphogenesis

To assess the developmental growth dynamics of the GL we analysed gut segments including the stomach, duodenum, pancreas and spleen between embryonic day 10.5 (e10.5) and postnatal day 0 (P0) in normal C57Bl/6 mice based on the signal from E-cadherin antibodies and endogenous tissue fluorescence for epithelium and mesenchyme respectively. The hereby-generated 3D time-line of pancreas organogenesis (Figure 1D–Q and Video S1, Video S2, Video S3, Video S4, Video S5, Video S6 and Video S7) revealed that the GL begins to form by perpendicular growth from the stalk of the DP epithelium around e13.5. During subsequent development, the GL epithelium colonizes a mesenchymal domain extending from the proximal DP mesenchyme overlying the pyloric sphincter and pyloric antrum (Figure 1G–J and N–Q and Video S4, Video S5, Video S6 and Video S7). Quantitative OPT analyses demonstrated that from e15.5 onwards, the GL epithelium constitutes more than 10% of the total pancreatic epithelial volume and that its growth rate is similar to that of the DP and VP (Figure 1B–C). Thus, the GL represents a significant portion of the pancreas that is formed by lateral growth from the stalk of the dorsal pancreatic anlage approximately four days later than the formation of the dorsal pancreatic bud around e9.5.

### GL morphogenesis is perturbed in mice displaying disturbed spleen development

The stomach and spleno-pancreatic mesenchyme constitute two separately inducible cell populations. During splenogenesis precursors located in the latter population expand in an anterior direction guided by signals originating from the anterior aspect of the stomach [11]. This process coincides with a condensation of the spleen mesenchyme across the length of the left side of the greater curvature of the stomach [8]. Our data reveal that growth of the GL epithelium is initiated at a stage when the DP mesenchyme and the developing spleen are morphologically distinct and leftward growth of both structures is well established. To elucidate the morphological events underlying the formation of the GL we focused our investigation on the gut-associated mesenchyme, before and around the time for initial GL formation (Figure 1L–O). From e11.5 onwards, a morphologically distinguishable spleen anlage can be recognized in the distal spleno-pancreatic mesenchyme (the spatial localization of the spleen anlage was verified by immunohistochemical analyses for Tlx1/Hox11 (Figure S1)). As development advances, spleen progenitors condense and move in an anterior direction over the greater curvature of the stomach. This process results in an apparent separation of the DP mesenchyme into two distinct domains, disjointed by a wedge of primordial spleen cells (Figure 1M). The hereby-formed perpendicular mesenchymal domain gradually becomes separated from the splenic primordium and the distal DP mesenchyme and at around e13.5 lateral growth from the stalk of the DP epithelium into this domain was observed (Figure 1N).

**Figure 2:**
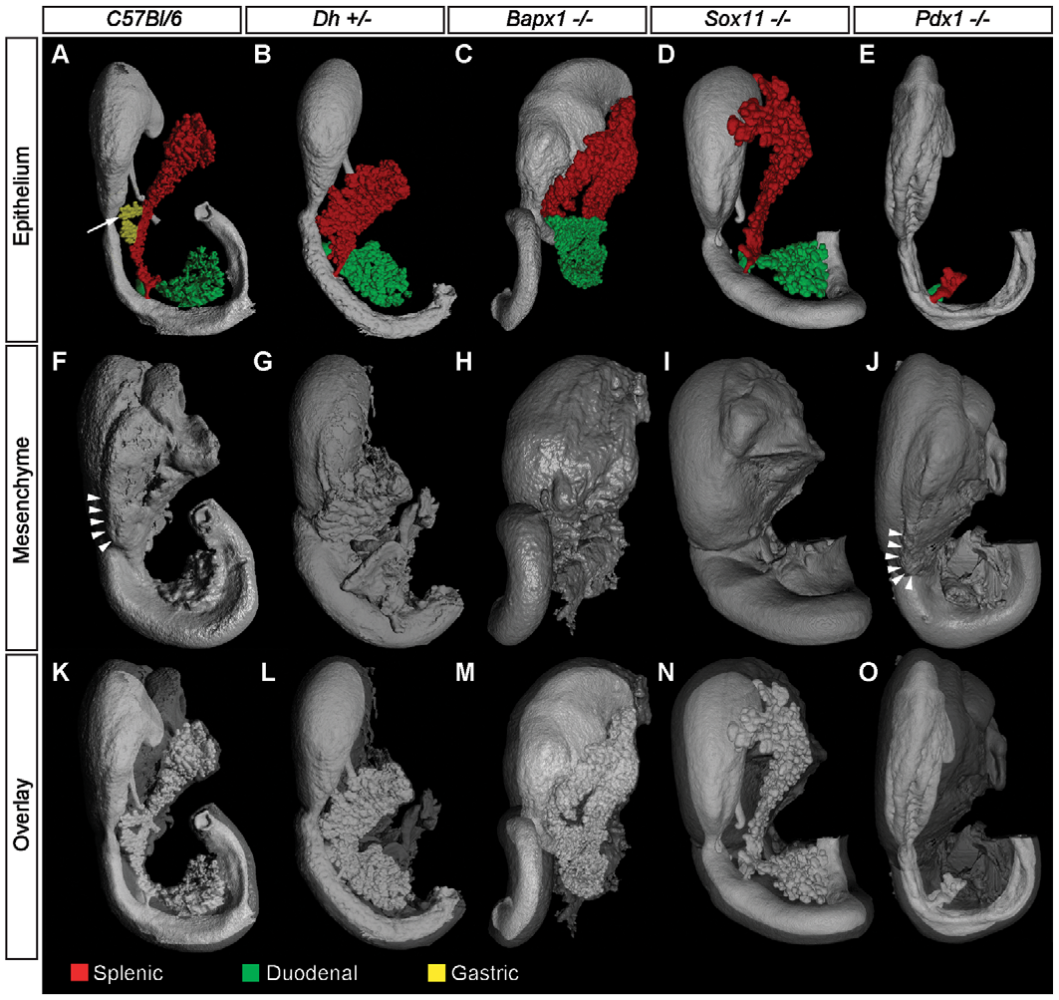
Disturbed splenogenesis perturbs formation of the gastric lobe mesenchymal domain. (**A** through **O**) OPT generated iso-surface reconstructions of the e14.5 pancreatic region from normal C57/Bl6 (**A**, **F**, **K**), *Dh+/−* (**B**, **G**, **L**), *Bapx1−/−* (**E**, **H**, **M**), *Sox11−/−* (**D**, **I**, **N**) and *Pdx1−/−* (**E**, **J**, **O**) mice. Reconstructions include the stomach, duodenum, spleen and pancreas and is based on the signal from; E-cadherin antibodies (epithelium – light grey, **A** to **E** and **K** to **O**) and the signal from tissue auto fluorescence (mesenchyme – dark grey, **F** to J and K to O). The incapacity to form a proper spleen primordium in *Dh+/−*, *Bapx1−/−* and *Sox11−/−* mice results in various degree of failure to separate the dorsal and gastric lobe mesenchyme. Thereby development of the entire dorsal epithelial region is perturbed (compare **B** to **D** with **A**). The gastric lobe is indicated by an arrow in (**A**). As indicated by mesenchymal morphogenesis in the Pdx1 mutant, formation of the GL mesenchymal domain is an autonomous event independent of pancreatic epithelial development (arrowheads, **F** and **J**). In (**A** to **G**) the dorsal, ventral and gastric lobes have been pseudocolored red, green and yellow respectively. The specimens are not depicted to scale.

Given the morphological indication of a role for spleen organogenesis in establishment of the GL mesenchyme we next investigated the impact of disturbed spleen development on GL formation. The transcriptional hierarchy governing spleen development is not fully understood. Available data, obtained primarily by assessments of knock out mice, suggest that at least two parallel genetic pathways are at play during early spleen development [12] and a number of mouse mutants displaying varying degrees of impaired spleen formation have been described. These include mice deficient for; *Tlx1(Hox11)* [13], [14], *Nkx2.3* [15], *Nkx3.2(Bapx1)* [16], [17], Tcf21*(POD1/Capsulin)* [18], *Wt1* [19], *Barx1* [20], *Sox11* [21], *Pbx1* [22] and the *Dh* mutant [17], [23]. Of these, Tlx1, Tcf21, Wt1, Nkx2.3, Pbx1, and Barx1 null mice have been demonstrated to form, at least initially, a defined spleen primordium. For example, in the Tlx1 mutants, the spleen primordium develops normally until e13.5 but fails to expand beyond this stage [13]. In contrast, *Dh+/−* mutants do not form a recognizable spleen primordium [17] and in *Bapx1−/−* embryos the spleen mesenchyme does not condense to form a morphologically distinct spleen anlage [8], [17]. Hence, these mutants display some of the most severe aberrations of spleen organogenesis described. OPT generated iso-surface renderings revealed that the GL epithelial domain was completely absent or severely truncated in *Dh+/−* mice at e14.5 and later stages (Figure 2 and data not shown). Although the *Bapx1−/−* GL phenotype is more difficult to assess due to an apparent looping of the duodenum during early development, we could similarly not detect the formation of a discernable GL in these mice (Figure 2). Analyses of mesenchymal morphogenesis in both mutants two days earlier, i.e. at e12.5, revealed that failure to form a distinct spleen anlage results in the inability to form two separately recognizable mesenchymal domains, corresponding to the GL mesenchyme and DP mesenchyme respectively (Figure S2). A thorough assessment of the developmental mechanisms responsible for asplenia in the Sox11 null mutant has not been presented. Our OPT data of mesenchymal morphology in the spleno-pancreatic region of Sox11 null mutants indicate that the initial formation of a recognizable spleen anlage does commence, albeit with partial phenotypic penetrance. Hence, at e12.5 Sox11 mice displayed essentially normal spleen primordia while at e14.5 the spleen morphology showed varied degree of perturbation (Figure 2). In further support of a role for proper spleen organogenesis in establishing the GL mesenchymal domain the degree of GL epithelial development varied in these mice, ranging from an observable truncation of the GL epithelium to essentially normal. In order to characterize the process of GL formation by an alternative approach, we attempted to establish an organ culture system for the purpose of *in vitro* manipulation. However, regardless of culture conditions (filter, matrigel and collagen cultures) we could not recapitulate normal gastric lobe morphogenesis with sufficient reproducibility, thus excluding the possibility for such assessments. As demonstrated in mice deficient for Pdx1(Ipf1), the morphogenesis of the DP mesenchyme is uncoupled from that of DP epithelium [7]. In those mutants, in which the pancreatic epithelium is growth arrested around e10.5, the spleen develops normally and the DP mesenchyme grows and develops morphologically and functionally independently of the DP epithelium [7]. Analyses of *Pdx1−/−* embryos revealed that similarly to the DP mesenchyme, the GL mesenchyme essentially occupies its normal space and position in the absence of a developing pancreatic epithelium. These data show that establishment of the GL mesenchyme, as well as the spatial information for its morphogenesis, is independent of pancreatic epithelial morphogenesis (arrowheads Figure 2J). Collectively, in support of the notion that spleen formation from within the mesenchyme overlying the dorsal aspect of the pancreas is required for formation of the GL mesenchymal domain, and thereby the GL epithelium; *Dh+/−*, *Bapx1−/−* and *Sox11−/−* mice all display varying degrees of impaired GL development.

### Gastric lobe formation appears independent on the mechanisms that direct L-R asymmetry during early spleno-pancreatic development

Leftward growth of the spleno-pancreatic (dorsal) region is mediated by a transient columnar mesodermally derived cell layer, the splanchnic mesodermal plate (SMP), which is under influence of the left-right genetic cascade. Leftward growth of the SMP itself appears to be driven by cell proliferation but it is also a source of growth factors such as FGF10, which is suggested to be a chemotactic factor for pancreatic growth [17]. To elucidate if similar mechanisms are at play also during GL formation we screened the mesenchyme surrounding the presumptive GL between e12.5–14.5. Hereby, we could neither detect a columnar cell layer analogous to the SMP nor increased mitotic activity or expression of FGF10 in the GL mesenchyme (Figure S3). The SMP is also suggested to play a role in the induction of the splenic mesenchyme [17]. *Bapx1−/−* mice form a SMP, although less pronounced, and in *Dh+/−* mice the structure fails to develop [17]. In *Sox11−/−* mutants however the SMP appears to form normally (Figure S3). Collectively, these results suggest that the mechanisms directing asymmetric growth of the DP are not involved in GL formation other than as a possible indirect result of a potential role for the SMP in spleen development [17].

### The gastric lobe displays a prolonged capacity to maintain markers for multipotent progenitor cells

Initial growth of the GL epithelium into the GL mesenchyme, around e13.5, coincides with an important hallmark of pancreas development. It was recently proposed that pancreas organogenesis is guided by multipotent progenitors that give rise to the endocrine, exocrine and ductal cell lineages of the pancreas. These cells, located in the tip of the branching DP, express Pdx1 and CPA1 but are negative for the exocrine marker Amylase. However, around e13.5 they begin to express markers for exocrine differentiation (Amylase) and subsequently give rise only to exocrine progeny [24]. The GL lobe contains all major pancreatic cell lineages. Consequently, unless the tip cells of the branching GL epithelium would maintain this multipotent potential for a longer time period than the DP epithelium from which they are derived, the GL-derived cell lineages would be likely to have a different descent. By performing triple labelling for Pdx1, CPA1 and Amylase at e12.5 to e15.5, in the ventral and dorsal pancreatic lobes we could verify that Pdx1^+^, CPA1^+^ and Amylase^−^ (Figure S4) cells were present in the dorsal and ventral lobes until e13.5 whereas at e14.5 the majority of the cells were Pdx1^+^, CPA1^+^ and Amylase^+^. The same set of markers applied to the GL at the corresponding stages suggested that the Pdx1^+^, CPA1^+^ and Amylase^−^ cells of this lobe were maintained until e14.5 (Figure S4). These results suggest that the cellular differentiation of the developing pancreas from a temporal point of view is not uniform, even between the dorsally derived lobes.

## Discussion

**Figure 3:**
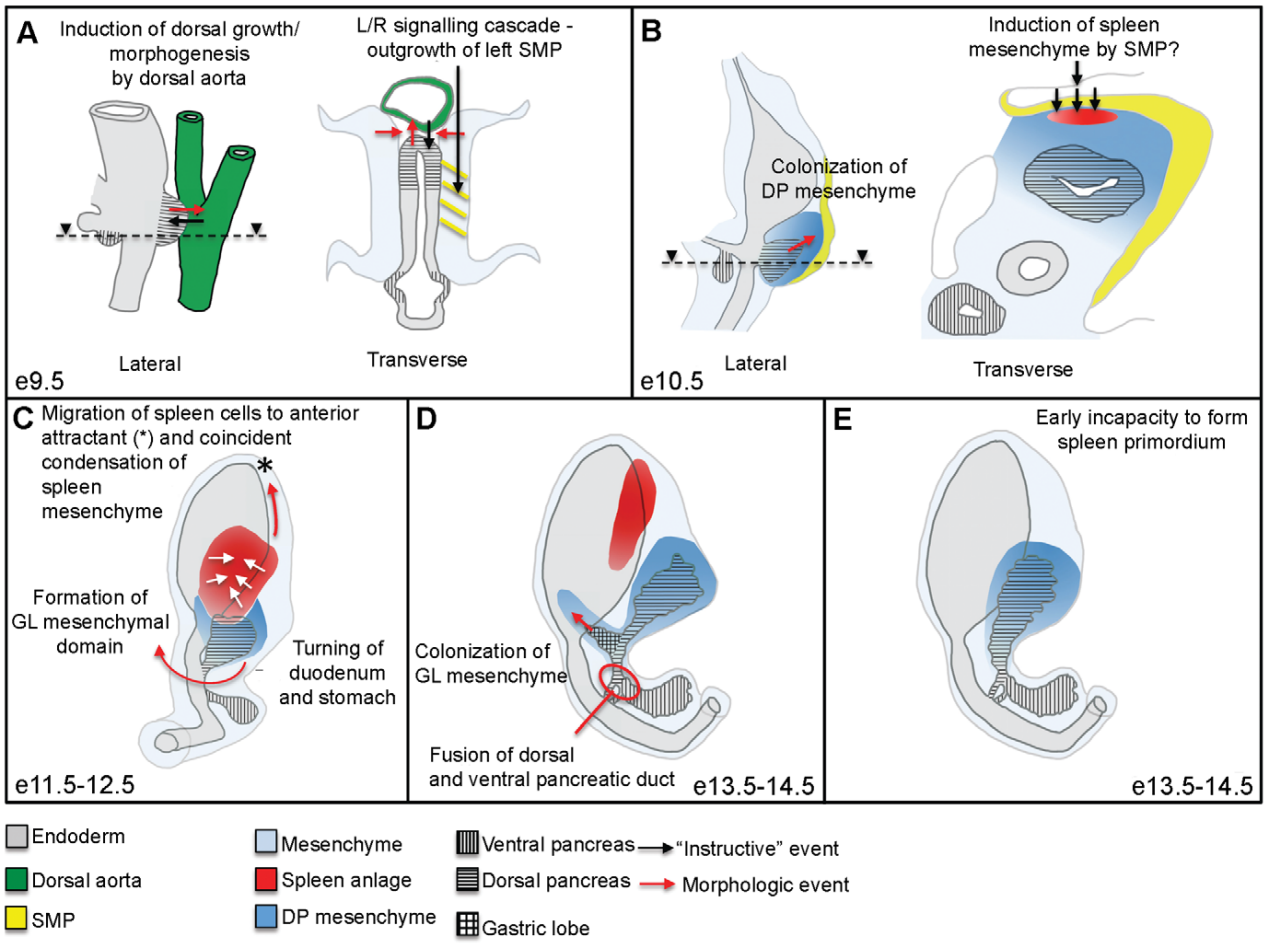
Development of the dorsal pancreatic region is dependent on sequential interaction with neighboring tissues. (**A**) The initial steps of pancreas development include the induction of the pancreatic anlage by signals originating from the notochord and dorsal aorta [31], [32] and are followed by the migration of splanchnic mesenchyme to cover the dorsal epithelium [28]. The ventral pancreas forms caudally to the presumptive liver anlage, distal from the cardiac mesoderm [33], [34], [35]. The left SMP is formed under influence of the L/R genetic cascade. (**B** to **D**) During subsequent development the DP and VP buds grow branch and differentiate as they invade the surrounding mesenchyme. (**B**) On the dorsal side, the SMP mediates leftward growth of the spleno-pancreatic region. (**C**) Spleen precursor cells migrate and condense (white arrows) over the left side of the greater curvature of the stomach and this process mediates the subdivision of the pancreatic mesenchyme into a dorsal and a gastric domain. (**D**) The GL mesenchymal domain provides a template for epithelial growth from the stalk of the DP epithelium. (**E**) In absence of proper spleen formation, the GL does not form and the morphology of the entire dorsal epithelium is perturbed.

The pancreatic epithelium is shaped by distinct stepwise cellular mechanisms to form a functional branching organ, and it was recently shown that the gross branch patterns show predictable trends during development [6], [25]. In this report we provide a 3D and quantitative description of the morphological events underlying normal pancreatic organogenesis in the mouse. Based on these data and analyses of mouse mutants displaying disturbed spleen development, we propose a model in which organogenesis of a neighbouring organ - the spleen - is required for normal morphogenesis of the dorsal pancreatic region and for the formation of the gastric pancreatic lobe in particular (Figure 3). In the examined mice mutants, we cannot rule out the possibility that the lack of gene function itself contribute to the perturbance of GL morphogenesis. However, given that the most severe splenic phenotype also coincides with the most pronounced perturbance of GL morphogenesis it seems reasonable that spleen formation is an important mediator of mesenchymal morphogenesis in the dorsal pancreatic region. Mesenchymal signals are important stimulators of pancreatic growth, branching and differentiation [26] and heterogeneous gene expression patterns have been described in the pancreas associated mesenchyme during different stages of development [6], [17]. However, the developmental significance of these observations is not fully understood. The question of whether unique signalling molecules and/or pathways are at play in the GL mesenchyme, which contribute to the formation of the GL epithelium therefore remains an open issue and is beyond the scope of this study. It is clear that the early steps of pancreatic epithelial development may commence with a variety of mesenchymal sources [27], [28], [29]. As suggested by this study, the spatial organization of the pancreatic mesenchyme, and the GL mesenchymal domain in particular, is mediated by spleen formation. Therefore, it is likely that this process, by itself is a key element in the establishment of the GL epithelial domain. As mentioned earlier, the auricle of the human pancreas has been suggested to be a reminiscent of the gastric lobe of the pancreas in rodents [5]. Although the location of this structure, as well as its close interaction with the gastroepiploic vessels, appear similar in both mice and humans, the issue of whether comparable morphological processes, as those described in this report, are at play during development of the human pancreas needs to be determined. Given the ethical and practical constraints associated with experimentation on human material, to investigate this issue will be a much challenging enterprise.

The herein described lobular heterogeneity regarding the temporal capacity to maintain Pdx1^+^, CPA1^+^ and Amylase^−^ cells may reflect a general delay in development of the GL. However, it also indicates that the local environment, even within the dorsal pancreatic region, influences the development of lobular characteristics of the pancreas. Hence, our data suggest that the dorsal, ventral and gastric pancreatic lobes together may provide an inherent system for comparative assessments of how cellular differentiation is coordinated within the pancreas. As to what extent this heterogeneity may influence other developmental or functional aspects of the pancreas remain a question for further investigation. Nevertheless, it seems likely that the close interplay between the splenic and pancreatic mesenchyme during early development described in this report must be taken into careful consideration in future assessments of the rodent pancreas.

## Material and Methods

### Ethics statement

All experiments were performed in compliance with the relevant national and institutional laws and guidelines. The study was approved by the Ethical Committee of Animal Research, Northern Sweden. Ethical approval ID A-8-2010.

### Animals

Mice were maintained as heterozygotes and/or bred and in the respective animal facility of: Umeå University, Sweden (Bapx1, Pdx1, C57Bl/6 (Taconic, Denmark)); MRC, Human Genetics Unit, Edinburgh, UK (Dh); Universität Erlangen Nuernburg, Germany (Sox11).

### Tissue preparation, immunohistochemistry and in situ hybridisation

Embryonic gut segments were dissected free, fixed in 4% paraformaldehyde and prepared for cryo-sectioning or whole-mount immunohistochemistry. Staining of cryosections was performed according to standard protocols. Cryosections with a thickness of 8 µm were obtained and blocked in 10% serum (from the same species in which the secondary antibody was derived) followed by antibody incubation. The adult pancreatic lobes were separated before OPT scanning. Antibody incubation time was reduced for embryonic tissues (24 h) and freeze-thawing was omitted. No bleaching of autoflourescence was performed for embryonic stages up to e14.5 [9]. Primary antibodies used were: Rabbit α-Hox11 (Santa Cruz Biotechnology), Rabbit α-Cpa1 (MediQip), Phalloidin (Molecular probes), Rabbit α-PH3 (Millipore), Rat α-E-cad (Zymed), Phalloidin-FITC (Sigma), Goat α-Pdx1 (Abcore), Guinea Pig α-Pdx1 (Abcam), Sheep α-Amy (Abcam). Primary antibodies were visualized with Alexa 488, 594 and 633-conjugated secondary antibodies (Molecular Probes). DIG-label in situ hybridization was performed according to standard protocols. FGF10 probe was made from full length cDNA of RIKEN clone 9430031A18.

### Optical projection tomography, volumetric quantification and image analysis

OPT analysis was performed essentially as described [9], [30]. Each specimen was scanned using the Bioptonics 3001 OPT scanner with a resolution of 1024×1024 pixels and reconstructed with the NRecon version 1.6.1.0 (Skyscan) software. Quantification of the pancreatic epithelium during development was made using the quantitation module for Volocity version 5.4.1 (Perkin Elmer). Reconstructed image stacks were digitally cropped to include the dorsal, ventral and gastric lobe epithelium respectively. The epithelial volumes were calculated by applying a “find objects by intensity” task to select voxels above a specified intensity. The intensity threshold value was manually determined for each image stack. A kernel filter (fine filter, 3×3 voxels) was used to remove background noise pixels with intensities above the selected threshold. All iso-surface reconstructions of embryonic gut segments were made using the visualization module for Volocity (Perkin Elmer), version 5.4.1. All measured volumes, adult and embryonic, were finally exported to the Excel 2007 (Microsoft Corporation, Redmond, Washington) software for statistical analysis (descriptive statistics). OPT images were exported as screenshots from iso-surface reconstructions in Volocity and processed in Photoshop CS2 version 9.0.2 (Adobe). All image adjustments were applied equally to entire images and occasional artefacts such as fibers or dust were digitally removed.

## Supporting Information

Figure S1 **OPT based assessments of Tlx1/Hox11 expression determining the spatial localization of the spleen primordium during embryonic development.** (**A** through **I**) OPT generated iso-surface reconstructions of gut segments including the stomach, duodenum, spleen and pancreas at e11.5 (**A**, **D**, **G**), e12.5 (**B**, **E**, **H**) and e13.5 (**C**, **F**, **I**) based on the signal from E-cadherin antibodies (epithelium, green in **A** to **C**), the signal from tissue autofluorescence (mesenchyme, **A** to **I**) and Tlx1 antibodies (spleen primordium, red in **A** to **F**). Compare with pseudo coloring in fig. 1 **I** to **K**.
(TIF)

Figure S2 **Early failure to form GL mesenchymal domain in** ***Dh +/−*** **and** ***Bapx1 −/−*** **mice.** OPT generated iso-surface reconstructions of gut segments including the stomach, duodenum, spleen and pancreas at e12.5 in normal C57/Bl6 (**A** and **D**), *Dh+/−* (**B** and **E**) and *Bapx1−/−* (**C** and **F**) mice. Reconstructions are based on the signal from E-cadherin antibodies (epithelium – light grey, **A** to **C**) and the signal from tissue autofluorescence (mesenchyme – dark grey, **A** to **F**). At e12.5, spleen condensation has mediated the formation of a GL mesenchymal domain lateral to the main bulk of dorsal pancreatic mesenchyme in wild-type mice (**A** and **E**). In *Dh +/−* mice (**B** and **F**), the complete absence of the spleen primordium prevents formation of a GL mesenchymal domain. In the *Bapx1 −/−* mice, morphogenesis of the GL mesenchymal domain is perturbed by the failure of the early spleen primordium to condense and dislocate from the pancreatic epithelium. The GL mesenchyme is indicated with arrowheads in **E**. Dorsal and ventral pancreatic epithelium have been pseudocolored red and green respectively in **A** to **C**. The pancreatic epithelial outline has been indicated with a white line in **D** to **F**. The specimens are not depicted to scale.
(TIF)

Figure S3 **The gastric lobe mesenchyme does not display characteristics analogous to those of the SMP during early leftward growth of the dorsal pancreas and spleen.** Sections of GL mesenchyme stained with phalloidin (red - **A**, **B**) and antibodies against phospho-histone H3 (red, **D**–**E**) and E-cadherin (green, **D**–**E**). (**C**) Iso-surface reconstruction of e12.5 gut segment based on tissue autoflourescence (mesenchyme) depicting the plane of section in (**A**–**B**, **D**–**E** and **G**–**I**). (F) Schematic representation of section plane shown in (**C**). Arrow indicates direction of GL growth. (**G**–**I**) In situ hybridization showing absence of FGF10 expression in GL mesenchyme between e12.5 and e14.5. (**J**–**K**) *Sox11 −/−* embryos display normal SMP (arrowheads) morphology at e10.5 as shown by phalloidin (green) and Pdx1staining (red). Abbreviations; dp, dorsal pancreas; glm, gastric lobe mesenchyme; ps, pyloric sphincter/posterior stomach epithelium; vp, ventral pancreas.
(TIF)

Figure S4 **The gastric lobe of the pancreas display a prolonged maintenance of markers for multipotent progenitor cells.** (**A** through **J**) Ventral (**A** to **D**), dorsal (**E** to **G**) and gastric (**I** and **J**) pancreas between e12.5 to 15.5 stained for Pdx1 (red), Carboxypeptidase A1 (CPA1, blue) and Amylase (Amy, green). At e14.5 the absolute majority of tip cells in the dorsal and ventral lobe have lost their progenitor potential and are; Pdx1^+^, CPA1^+^, Amy^+^ (arrows in **C** and **G**). In contrast, the gastric lobe tip cells are Pdx1^+^, CPA1^+^, Amy^−^ at the same stage and display an expression profile similar to the dorsal and ventral pancreas at e12.5 (arrowheads in **I**).
(TIF)

Video S1 Pancreatic epithelial and mesenchymal morphology at e10.5. OPT generated movie sequence depicting a gut segment including the stomach, duodenum, lung and pancreas with associated mesenchyme. Volume rendering of the mesenchyme is based on the signal from tissue autofluorescence (grey). Iso-surface rendering of the epithelium is based on the signal from E-cadherin antibody staining (yellow). At e10.5, the initial evaginations of the foregut epithelium have developed into distinct ventral and dorsal pancreatic buds.
(MP4)

Video S2 **Pancreatic epithelial and mesenchymal morphology at e11.5.** OPT generated movie sequence depicting a gut segment including the stomach, duodenum and pancreas with associated mesenchyme. Volume rendering of the mesenchyme is based on the signal from tissue autofluorescence (grey). Iso-surface rendering of the epithelium is based on the signal from E-cadherin antibody staining (yellow). At this stage the spleen anlage is morphologically recognizable in the mesenchyme covering the dorsal aspect of the pancreas.
(MP4)

Video S3 **Pancreatic epithelial and mesenchymal morphology at e12.5.** OPT generated movie sequence depicting a gut segment including the stomach, duodenum and pancreas with associated mesenchyme. Volume rendering of the mesenchyme is based on the signal from tissue autofluorescence (grey). Iso-surface rendering of the epithelium is based on the signal from E-cadherin antibody staining (yellow). As the stomach and duodenum rotate during development the dorsal and ventral pancreas starts to become positioned on the same side. The spleen anlage is morphologically distinct and starts to become separated from the dorsal pancreatic mesenchyme.
(MP4)

Video S4 **Pancreatic epithelial and mesenchymal morphology at e13.5.** OPT generated movie sequence depicting a gut segment including the stomach, duodenum and pancreas with associated mesenchyme. Volume rendering of the mesenchyme is based on the signal from tissue autofluorescence (grey). Iso-surface rendering of the epithelium is based on the signal from E-cadherin antibody staining (yellow). At this stage lateral growth from the stalk of the dorsal pancreatic epithelium into gastric lobe mesenchymal domain is observed. Note the fusion of the dorsal and ventral pancreatic ducts at some distance from the duodenum.
(MP4)

Video S5 **Pancreatic epithelial and mesenchymal morphology at e14.5.** OPT generated movie sequence depicting a gut segment including the stomach, duodenum and pancreas with associated mesenchyme. Volume rendering of the mesenchyme is based on the signal from tissue autofluorescence (grey). Iso-surface rendering of the epithelium is based on the signal from E-cadherin antibody staining (yellow). At this stage the gastric lobe is present as a clearly distinguishable epithelial domain overlying the pyloric sphincter.
(MP4)

Video S6 **Pancreatic epithelial and mesenchymal morphology at e15.5.** OPT generated movie sequence depicting a gut segment including the stomach, duodenum and pancreas with associated mesenchyme. Volume rendering of the mesenchyme is based on the signal from tissue autofluorescence (grey). Iso-surface rendering of the epithelium is based on the signal from E-cadherin antibody staining (yellow). From this stage onwards the gastric lobe constitutes more than 10% of the overall pancreatic epithelial volume and covers also the pyloric antrum.
(MP4)

Video S7 **Pancreatic epithelial and mesenchymal morphology at e17.5.** OPT generated movie sequence depicting a gut segment including the stomach, duodenum and pancreas with associated mesenchyme. Volume rendering of the mesenchyme is based on the signal from tissue autofluorescence (grey). Iso-surface rendering of the epithelium is based on the signal from E-cadherin antibody staining (yellow). At this stage the gastric lobe essentially occupies its adult position and shape.
(MP4)

## References

[1] Gittes GK (2009). Developmental biology of the pancreas: a comprehensive review.. Dev Biol.

[2] Pan FC, Wright C (2011). Pancreas organogenesis: From bud to plexus to gland.. Dev Dyn.

[3] Slack JM (1995). Developmental biology of the pancreas.. Development.

[4] Bock P, Abdel-Moneim M, Egerbacher M (1997). Development of pancreas.. Microsc Res Tech.

[5] Hagai H (2003). Configurational anatomy of the pancreas: its surgical relevance from ontogenetic and comparative-anatomical viewpoints.. J Hepatobiliary Pancreat Surg.

[6] Villasenor A, Chong DC, Henkemeyer M, Cleaver O (2010). Epithelial dynamics of pancreatic branching morphogenesis.. Development.

[7] Ahlgren U, Jonsson J, Edlund H (1996). The morphogenesis of the pancreatic mesenchyme is uncoupled from that of the pancreatic epithelium in IPF1/PDX1-deficient mice.. Development.

[8] Asayesh A, Sharpe J, Watson RP, Hecksher-Sorensen J, Hastie ND (2006). Spleen versus pancreas: strict control of organ interrelationship revealed by analyses of Bapx1−/− mice.. Genes Dev.

[9] Sand FW, Hornblad A, Johansson JK, Loren C, Edsbagge J (2011). Growth-limiting role of endothelial cells in endoderm development.. Dev Biol.

[10] Sharpe J, Ahlgren U, Perry P, Hill B, Ross A (2002). Optical projection tomography as a tool for 3D microscopy and gene expression studies.. Science.

[11] Burn SF, Boot MJ, de Angelis C, Doohan R, Arques CG (2008). The dynamics of spleen morphogenesis.. Dev Biol.

[12] Brendolan A, Rosado MM, Carsetti R, Selleri L, Dear TN (2007). Development and function of the mammalian spleen.. Bioessays.

[13] Dear TN, Colledge WH, Carlton MB, Lavenir I, Larson T (1995). The Hox11 gene is essential for cell survival during spleen development.. Development.

[14] Roberts CW, Shutter JR, Korsmeyer SJ (1994). Hox11 controls the genesis of the spleen.. Nature.

[15] Pabst O, Zweigerdt R, Arnold HH (1999). Targeted disruption of the homeobox transcription factor Nkx2–3 in mice results in postnatal lethality and abnormal development of small intestine and spleen.. Development.

[16] Lettice LA, Purdie LA, Carlson GJ, Kilanowski F, Dorin J (1999). The mouse bagpipe gene controls development of axial skeleton, skull, and spleen.. Proc Natl Acad Sci U S A.

[17] Hecksher-Sorensen J, Watson RP, Lettice LA, Serup P, Eley L (2004). The splanchnic mesodermal plate directs spleen and pancreatic laterality, and is regulated by Bapx1/Nkx3.2.. Development.

[18] Lu J, Chang P, Richardson JA, Gan L, Weiler H (2000). The basic helix-loop-helix transcription factor capsulin controls spleen organogenesis.. Proc Natl Acad Sci U S A.

[19] Herzer U, Crocoll A, Barton D, Howells N, Englert C (1999). The Wilms tumor suppressor gene wt1 is required for development of the spleen.. Curr Biol.

[20] Kim BM, Miletich I, Mao J, McMahon AP, Sharpe PA (2007). Independent functions and mechanisms for homeobox gene Barx1 in patterning mouse stomach and spleen.. Development.

[21] Sock E, Rettig SD, Enderich J, Bosl MR, Tamm ER (2004). Gene targeting reveals a widespread role for the high-mobility-group transcription factor Sox11 in tissue remodeling.. Mol Cell Biol.

[22] Brendolan A, Ferretti E, Salsi V, Moses K, Quaggin S (2005). A Pbx1-dependent genetic and transcriptional network regulates spleen ontogeny.. Development.

[23] Green MC (1967). A defect of the splanchnic mesoderm caused by the mutant dominant hemimelia in the mouse.. Dev Biol.

[24] Zhou Q, Law AC, Rajagopal J, Anderson WJ, Gray PA (2007). A multipotent progenitor domain guides pancreatic organogenesis.. Developmental Cell.

[25] Kesavan G, Sand FW, Greiner TU, Johansson JK, Kobberup S (2009). Cdc42-mediated tubulogenesis controls cell specification.. Cell.

[26] Kim SK, Hebrok M (2001). Intercellular signals regulating pancreas development and function.. Genes Dev.

[27] Ahlgren U, Pfaff SL, Jessell TM, Edlund T, Edlund H (1997). Independent requirement for ISL1 in formation of pancreatic mesenchyme and islet cells.. Nature.

[28] Wessells NK, Cohen JH (1967). Early pancreas organogenesis: morphogenesis, tissue interactions and mass effects.. Dev Biol.

[29] Golosow N, Grobstein C (1962). Epitheliomesrenchymal interaction in pancreatic morphogenesis.. Dev Biol.

[30] Alanentalo T, Asayesh A, Morrison H, Loren CE, Holmberg D (2007). Tomographic molecular imaging and 3D quantification within adult mouse organs.. Nat Methods.

[31] Yoshitomi H, Zaret KS (2004). Endothelial cell interactions initiate dorsal pancreas development by selectively inducing the transcription factor Ptf1a.. Development.

[32] Lammert E, Cleaver O, Melton D (2001). Induction of pancreatic differentiation by signals from blood vessels.. Science.

[33] Bort R, Martinez-Barbera JP, Beddington RS, Zaret KS (2004). Hex homeobox gene-dependent tissue positioning is required for organogenesis of the ventral pancreas.. Development.

[34] Deutsch G, Jung J, Zheng M, Lora J, Zaret KS (2001). A bipotential precursor population for pancreas and liver within the embryonic endoderm.. Development.

[35] Wandzioch E, Zaret KS (2009). Dynamic signaling network for the specification of embryonic pancreas and liver progenitors.. Science.

