# Supplementary figures and images for: Impaired Spleen Formation Perturbs Morphogenesis of the Gastric Lobe of the Pancreas
Source: PLoS One. 2011 Jun 30;6(6):e21753. doi: 10.1371/journal.pone.0021753 (PMC3128080; doi:10.1371/journal.pone.0021753)

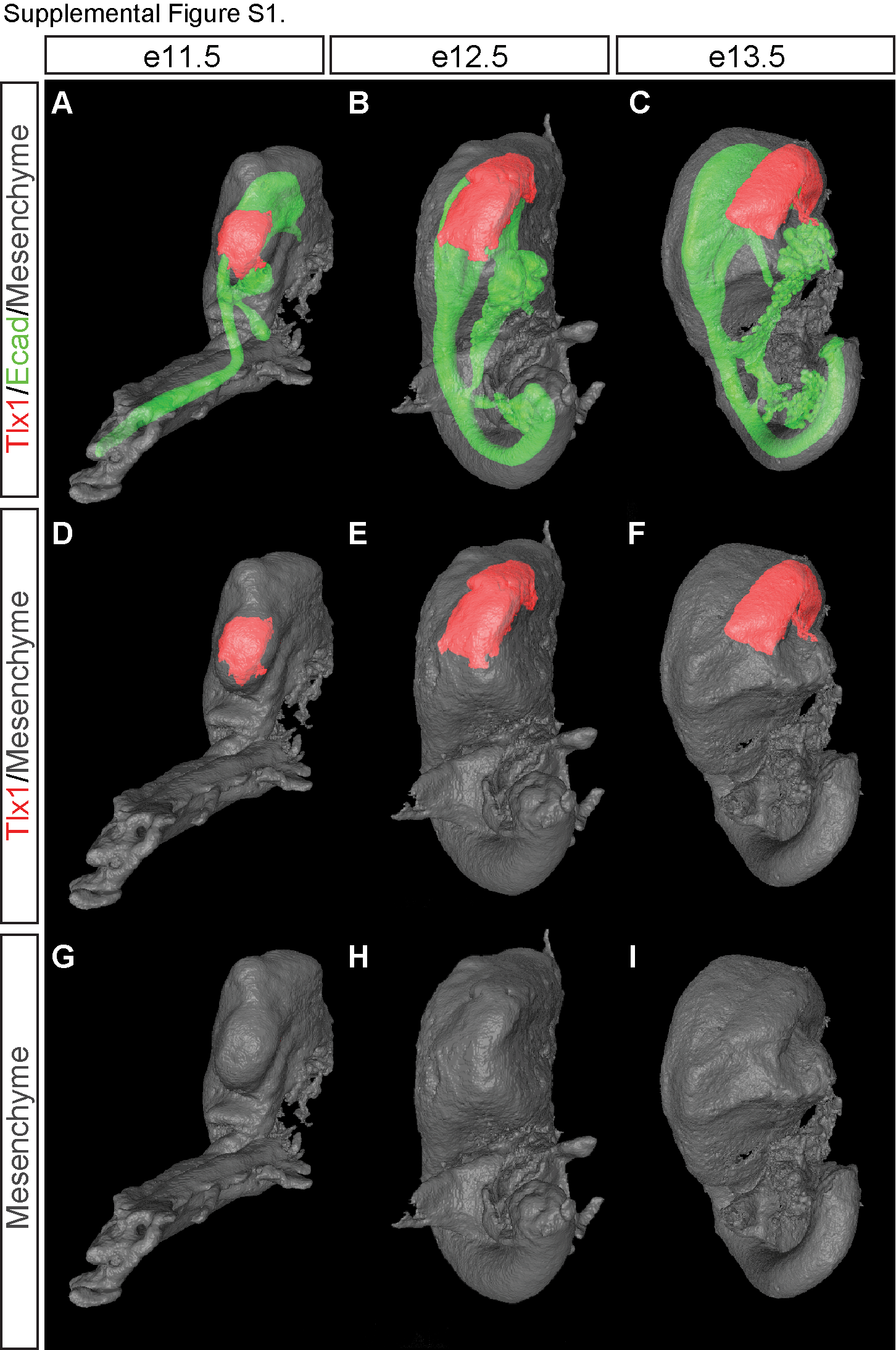

Supplement: Figure S1 — OPT based assessments of Tlx1/Hox11 expression determining the spatial localization of the spleen primordium during embryonic development. (A through I) OPT generated iso-surface reconstructions of gut segments including the stomach, duodenum, spleen and pancreas at e11.5 (A, D, G), e12.5 (B, E, H) and e13.5 (C, F, I) based on the signal from E-cadherin antibodies (epithelium, green in A to C), the signal from tissue autofluorescence (mesenchyme, A to I) and Tlx1 antibodies (spleen primordium, red in A to F). Compare with pseudo coloring in fig. 1 I to K. (TIF) [file pone.0021753.s001.tif]

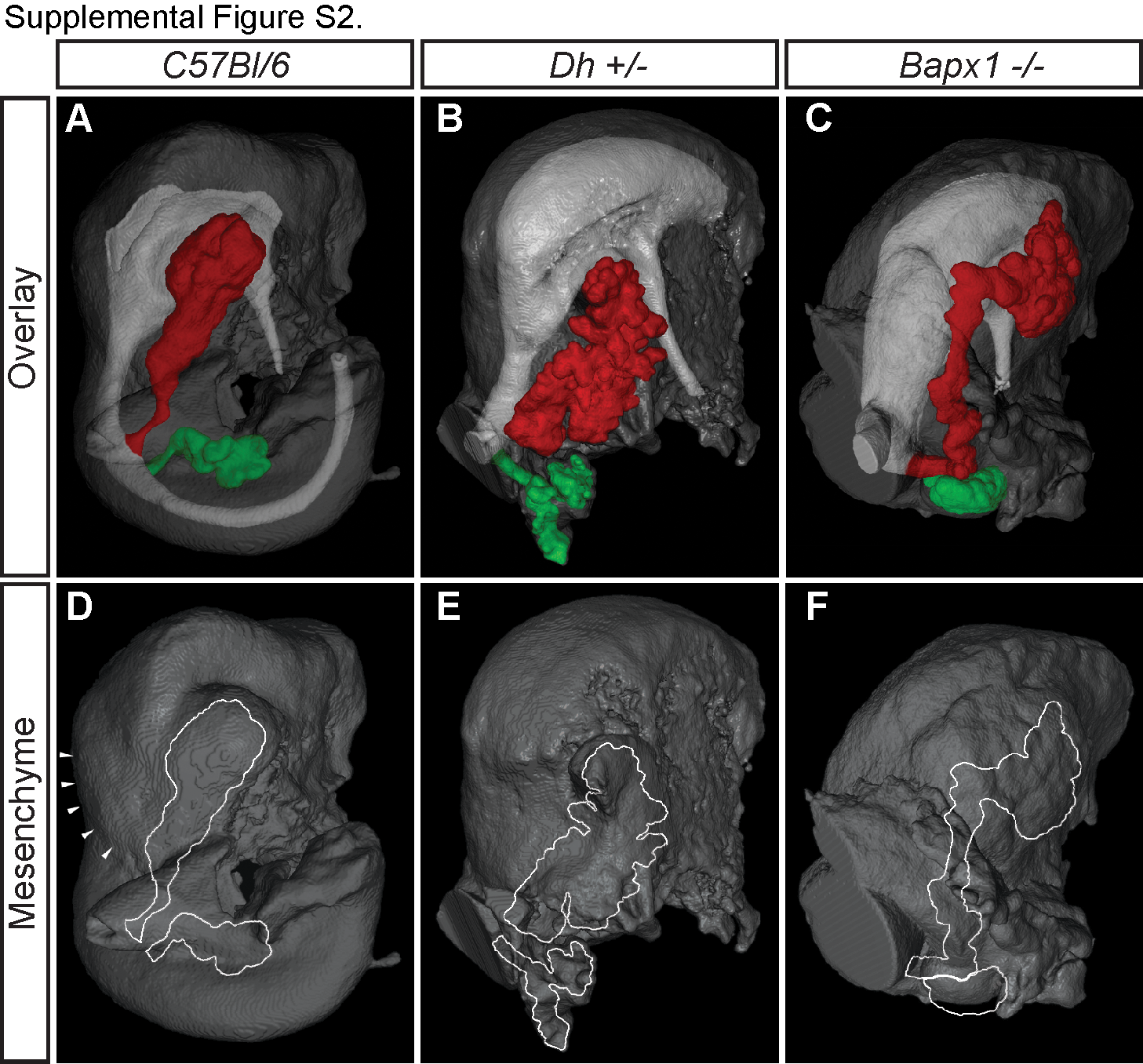

Supplement: Figure S2 — Early failure to form GL mesenchymal domain in Dh +/− and Bapx1 −/− mice. OPT generated iso-surface reconstructions of gut segments including the stomach, duodenum, spleen and pancreas at e12.5 in normal C57/Bl6 (A and D), Dh+/− (B and E) and Bapx1−/− (C and F) mice. Reconstructions are based on the signal from E-cadherin antibodies (epithelium – light grey, A to C) and the signal from tissue autofluorescence (mesenchyme – dark grey, A to F). At e12.5, spleen condensation has mediated the formation of a GL mesenchymal domain lateral to the main bulk of dorsal pancreatic mesenchyme in wild-type mice (A and E). In Dh +/− mice (B and F), the complete absence of the spleen primordium prevents formation of a GL mesenchymal domain. In the Bapx1 −/− mice, morphogenesis of the GL mesenchymal domain is perturbed by the failure of the early spleen primordium to condense and dislocate from the pancreatic epithelium. The GL mesenchyme is indicated with arrowheads in E. Dorsal and ventral pancreatic epithelium have been pseudocolored red and green respectively in A to C. The pancreatic epithelial outline has been indicated with a white line in D to F. The specimens are not depicted to scale. (TIF) [file pone.0021753.s002.tif]

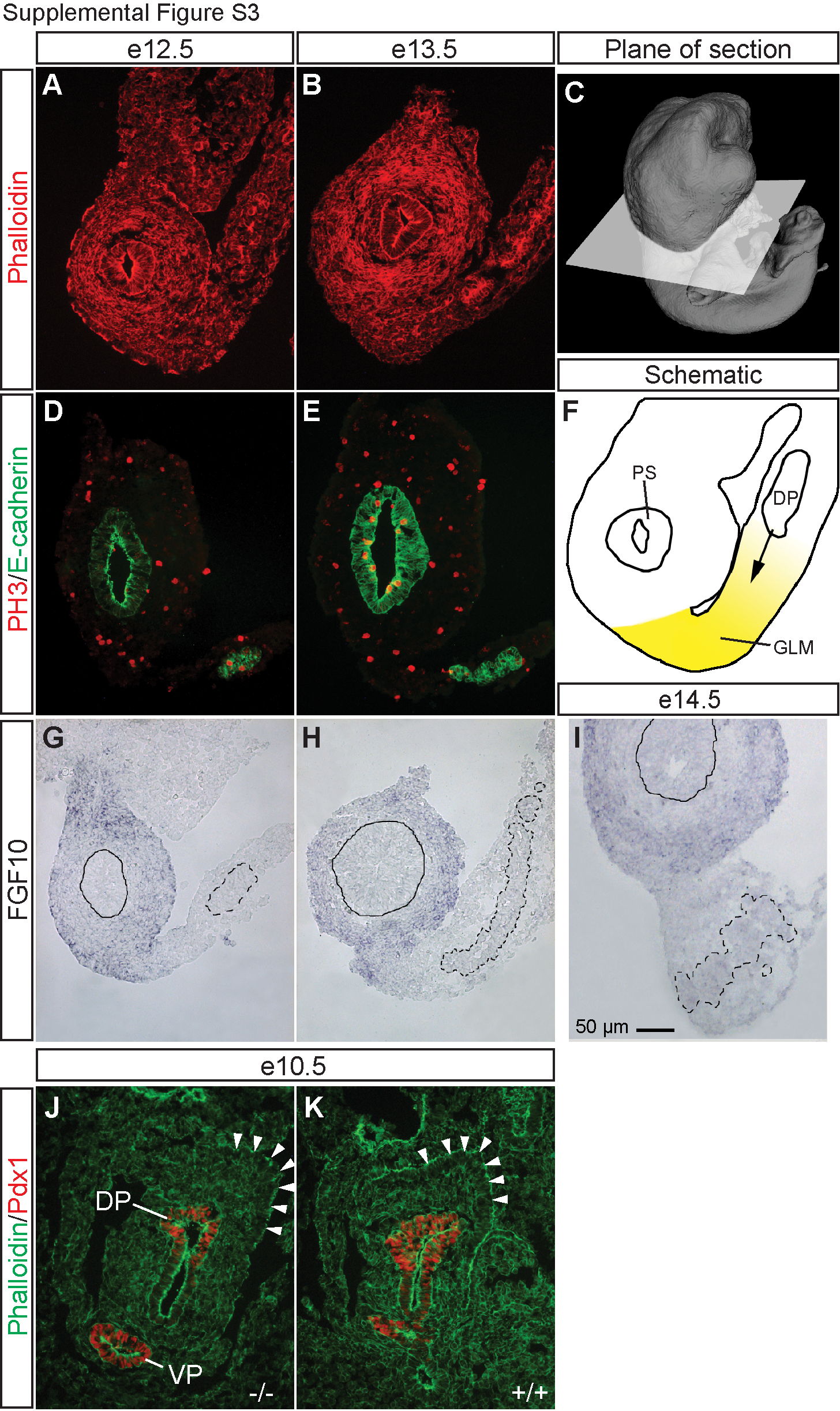

Supplement: Figure S3 — The gastric lobe mesenchyme does not display characteristics analogous to those of the SMP during early leftward growth of the dorsal pancreas and spleen. Sections of GL mesenchyme stained with phalloidin (red - A, B) and antibodies against phospho-histone H3 (red, D–E) and E-cadherin (green, D–E). (C) Iso-surface reconstruction of e12.5 gut segment based on tissue autoflourescence (mesenchyme) depicting the plane of section in (A–B, D–E and G–I). (F) Schematic representation of section plane shown in (C). Arrow indicates direction of GL growth. (G–I) In situ hybridization showing absence of FGF10 expression in GL mesenchyme between e12.5 and e14.5. (J–K) Sox11 −/− embryos display normal SMP (arrowheads) morphology at e10.5 as shown by phalloidin (green) and Pdx1staining (red). Abbreviations; dp, dorsal pancreas; glm, gastric lobe mesenchyme; ps, pyloric sphincter/posterior stomach epithelium; vp, ventral pancreas. (TIF) [file pone.0021753.s003.tif]

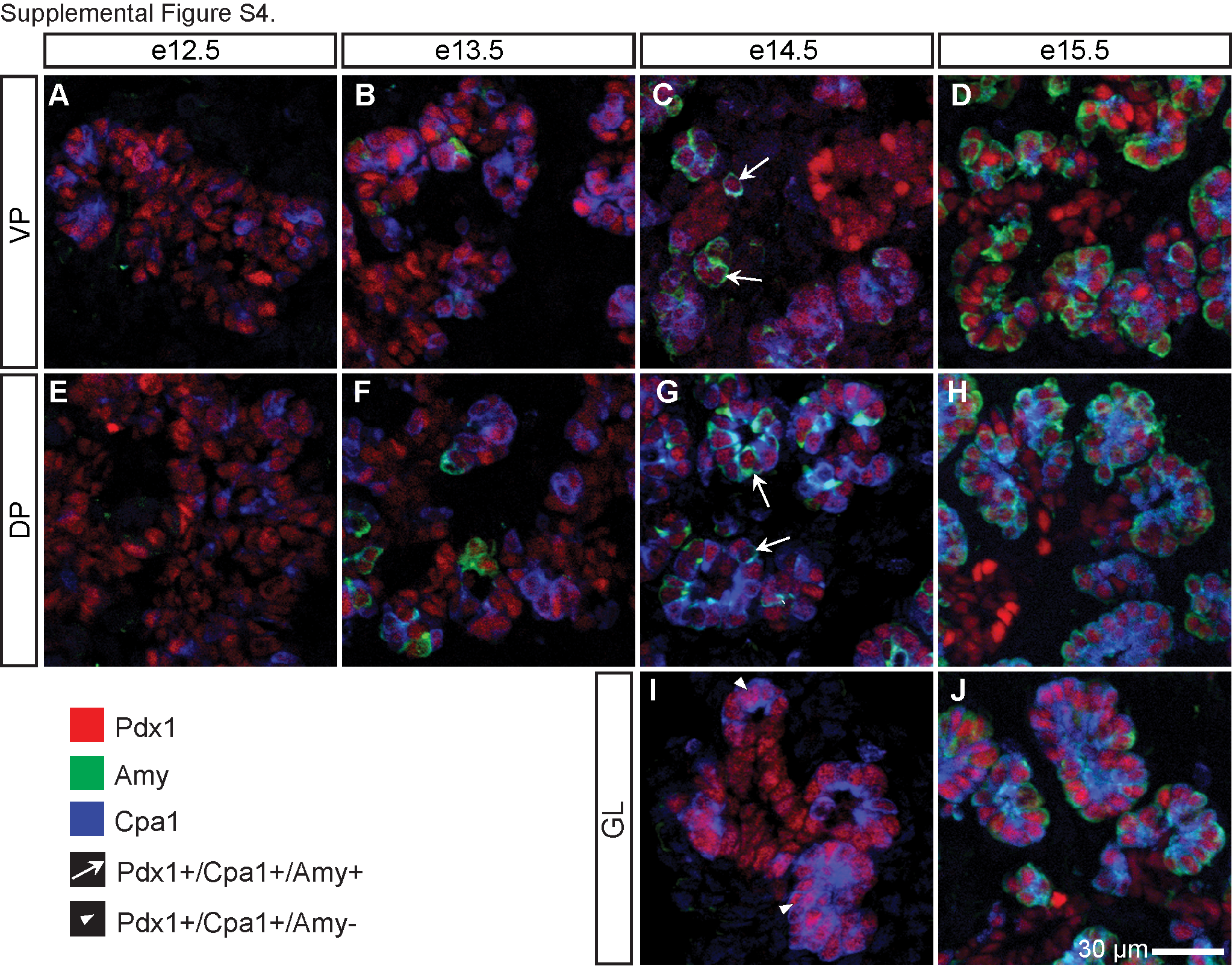

Supplement: Figure S4 — The gastric lobe of the pancreas display a prolonged maintenance of markers for multipotent progenitor cells. (A through J) Ventral (A to D), dorsal (E to G) and gastric (I and J) pancreas between e12.5 to 15.5 stained for Pdx1 (red), Carboxypeptidase A1 (CPA1, blue) and Amylase (Amy, green). At e14.5 the absolute majority of tip cells in the dorsal and ventral lobe have lost their progenitor potential and are; Pdx1+, CPA1+, Amy+ (arrows in C and G). In contrast, the gastric lobe tip cells are Pdx1+, CPA1+, Amy− at the same stage and display an expression profile similar to the dorsal and ventral pancreas at e12.5 (arrowheads in I). (TIF) [file pone.0021753.s004.tif]
